# Back Interface Passivation for Efficient Low-Bandgap Perovskite Solar Cells and Photodetectors

**DOI:** 10.3390/nano12122065

**Published:** 2022-06-15

**Authors:** Jiayu Lu, Huayang Wang, Tingbing Fan, Dong Ma, Changlei Wang, Shaolong Wu, Xiaofeng Li

**Affiliations:** 1 Collaborative Innovation Center of Suzhou Nano Science and Technology, Key Lab of Advanced Optical Manufacturing Technologies of Jiangsu Province & Key Lab of Modern Optical Technologies of Education Ministry of China, School of Optoelectronic Science and Engineering, Soochow University, Suzhou 215006, China; 20205239017@stu.suda.edu.cn (J.L.); 20204239018@stu.suda.edu.cn (H.W.); 20215239025@stu.suda.edu.cn (T.F.); xfli@suda.edu.cn (X.L.); 2 School of Rail Transportation, Soochow University, Suzhou 215137, China

**Keywords:** low-bandgap perovskite, interface passivation, perovskite solar cells, self-powered photodetectors, visible light communication

## Abstract

Low-bandgap (E_g_~1.25 eV) mixed tin-lead (Sn-Pb) perovskites are promising candidates for efficient solar cells and self-powered photodetectors; however, they suffer from huge amounts of defects due to the unintentional p-type self-doping. In this work, the synergistic effects of maltol and phenyl-C61-butyric acid methyl ester (PCBM) were achieved to improve the performance of low-bandgap perovskite solar cells (PSCs) and unbiased perovskite photodetectors (PPDs) by passivating the defects and tuning charge transfer dynamics. Maltol eliminated the Sn-related traps in perovskite films through a strong metal chelating effect, whereas PCBM elevated the built-in electric potential and thus improved voltage through the spike energy alignment. Combining both advantages of maltol and PCBM, high-quality perovskite films were obtained, enabling low-bandgap PSCs with the best efficiency of 20.62%. Moreover, the optimized PSCs were further applied as self-powered PPDs in a visible light communication system with a response time of 0.736 μs, presenting a satisfactory audio transmission capability.

## 1. Introduction

Organic-inorganic hybrid perovskite materials have unique optoelectronic advantages and are excellent candidates for high-performance solar cells and self-powered photodetectors [1,2,3,4,5]. Perovskite materials have a wide bandgap tunability ranging from 1.2 electron volt (eV) to 2.3 eV by compositional engineering. Pure-lead (Pb)-based perovskites usually have the bandgaps over 1.5 eV, far away from the optimal bandgap for single-junction solar cells [6,7]. Mixed tin (Sn)-Pb perovskites have bandgaps as low as ~1.2 eV due to the bowling effect, and the corresponding performance of low-bandgap PSCs has been pushed to 23.6% recently [8]. Moreover, high performance single-junction PSCs can also work well as self-powered photodetectors (PDs). Low-bandgap mixed Sn-Pb based perovskites PDs (PPDs) have a substantial optoelectronic response from ultraviolet to the near-infrared (NIR) bands [9,10,11,12,13,14].

Low-bandgap mixed Sn-Pb-based PSCs and PPDs have been intensively explored in recent years and gained prosperous developments. However, the easy oxidization of Sn^2+^ to Sn^4+^ makes the Sn-based perovskites vulnerable to ambient conditions [15]. The presence of Sn^4+^ in the perovskites leads to heavily p-type self-doping as well as numerous defects, such as Sn vacancies [16]. Various strategies have been proposed to suppress the defects in mixed Sn-Pb perovskites, such as antioxidants and oxygen scavengers in the perovskite precursor and films. Sn powders, SnF_2_, 4-trifluoromethyl-phenylammonium (CF3-PA), and pyrazine have been reported to reduce the density of trap states [17,18,19]. Maltol, a kind of green chemical that is usually used as a food additive, has also been proposed to passivate the traps and suppress Sn oxidizations in the Sn-based perovskites due to its strong metal chelating properties [20]. In addition, energy level alignment also plays a crucial role in manipulating the charge transfer dynamics in PSCs and PPDs [21]. phenyl-C61-butyric acid methyl ester (PCBM) has been employed as the energy spike interlayer in low-bandgap PSCs to elevate the built-in electric field [22], thus leading to relatively higher open-circuit voltage (*V*_oc_) [23].

In this work, we systematically investigated the synergistic effects of maltol post-treatment and PCBM interlayers on the quality of perovskite film and the corresponding device performances of low-bandgap mixed Sn-Pb perovskites. Maltol can bind on uncoordinated Sn^2+^ on the film surface, inhibiting the Sn oxidation and reducing the formation of Sn vacancies; and PCBM can slightly elevate the energy level of electron transporting layers with an upshifted Fermi level, leading to a higher built-in potential. As a result, optimized PSCs with a power conversion efficiency (PCE) of 20.62% were achieved. More importantly, our PSCs can also work well as self-powered PPDs, with improved response time, presenting satisfactory applications for audio transmission in a visible light communications system.

## 2. Materials and Methods

### 2.1. Materials

N, N-dimethylformamide (DMF, anhydrous), dimethyl sulfoxide (DMSO, anhydrous), toluene (anhydrous) chlorobenzene (CB, anhydrous), lead thiocyanate (Pb(SCN)_2_, 95%), tin powders, and tin fluoride (SnF_2_) were purchased from Sigma-Aldrich (St. Louis, MO, USA). Formamidinium iodide (FAI) was purchased from Greatcell Solar Company (New South Wales, Australia). Methylammonium iodide (MAI), lead iodide (PbI_2_), methylammonium bromine (MABr), and maltol were purchased from Xi’an polymer company. Tin iodide (SnI_2_) was purchased from Advanced Election Technology (Yingkou, China). Lead(II) bromide (PbBr_2_) was purchased from Alfa Aesar (Ward Hill, MA, USA). Poly(3,4-ethylenedioxythiophene): polystyrene sulfonate (PEDOT:PSS) aqueous solution (Clevious PVP AI 4083) was purchased from Heraeus Co., Ltd. (Hanau, Germany). C_60_ was purchased from NanoC company (Westwood, CA, USA). Bathocuproine (BCP) was purchased from Jilin OLED company (Jilin, China). These chemicals were used as obtained without further purification.

### 2.2. Perovskite Precursor Solution

The precursor of low-bandgap perovskite (FASnI_3_)_0.6_(MAPbI_3_)_0.37_(MAPbBr_3_)_0.03_ was prepared by mixing FASnI_3_, MAPbI_3,_ and MAPbBr_3_ solutions with a volume ratio of 0.6:0.37:0.03. For FASnI_3_ solution (1.57 M), 172 mg FAI, 372 mg SnI_2_ and 7.8 mg SnF_2_ (5 mol % relative to SnI_2_) were dissolved in a 636 µL mixed solution of DMF and DMSO (*v*/*v* = 2:1). Tin power (5 mg) was added in FASnI_3_ solution to avoid the Sn^2+^ oxidization. MAPbI_3_ solution (1.57 M) was peppered by dissolving 159 mg MAI, 461 mg PbI_2,_ and 13.9 mg Pb(SCN)_2_ in 565 µL DMF and 71 µL DMSO. In addition, 112 mg MABr and 367 mg PbBr_2_ were dissolved in 565 µL DMF and 71 µL DMSO for MAPbBr_3_ (1.57 M) solution. All perovskite precursors were filtered with a 0.45 μm filter. The final low-bandgap perovskite precursor was mixed for two hours before use.

### 2.3. Maltol and PCBM Solutions

Maltol was dissolved in toluene with a concentration of 1 mg/mL. PCBM powder was dissolved in CB with a concentration of 10 mg/mL. All solutions were filtered before use.

### 2.4. Device Fabrication

The ITO glass substrates were cleaned by ultrasonication with detergent, acetone, and an alcohol bath for 15 min sequentially. PEDOT:PSS solution was spin-coated on the ITO substrates at 6000 rpm for 40 s, and then dried at 150 °C for 30 min. Then, the hole transport layer (HTL)-coated substrates were transferred into a glovebox. Perovskite films were deposited on the HTLs at 4000 rpm for 60 s. Diethyl ether (650 µL), as an antisolvent, was dripped on the film surface in the first 5 s during spinning. The obtained film was then annealed at 65 °C for 3 min and 100 °C for 7 min. Maltol were spin-coated on perovskite film at 4000 rpm for 20 s, and then heated at 65 °C for 2 min. PCBM were spin-coated on perovskite film at 2000 rpm for 30 s then dried at 65 °C for 10 min. The whole spin coating and heating processes were conducted in a glovebox with H_2_O and O_2_ concentrations lower than 0.1 ppm. Finally, C_60_ (15 nm), BCP (8 nm), and Ag (100 nm) were sequentially evaporated on the top of perovskite films.

### 2.5. Material Characterizations and Device Performance Measurements

Scanning electron microscopy (SEM) images were obtained using field emission scanning electron microscopy (Zeiss Gemini sigma 300, Oberkochen, Germany). Surface roughness images were measured using atomic force microscope (Bruker Dimension Icon., Berlin, Germany) with 5 µm × 5 µm areas. Photoluminescence spectroscopy (PL) measurements use a Monochromatic Xe lamp as the excitation source with a 520 nm peak wavelength. For the time-resolved photoluminescence spectroscopy (TRPL) measurement, a 405 nm picosecond laser was used as the excitation source. Kelvin probe force microscopy (KPFM) was measured to determine the surface work functions of perovskite films. Transmission and Reflection spectrum were measured using an ultraviolet-visible-NIR spectrophotometer (PerkinElmer, Lamda 1050S, Waltham, MA, USA).

The current versus voltage (*J-V*) characteristics were measured using source meter (Keithley 2400, Biferton, Melrose, MA, USA) under the illumination of the simulated AM 1.5 G light (SS-F5-3A, Enlitech, Taiwan, China). The light intensity was calibrated using a reference silicon solar cell. The *V*_oc_, short-circuit current density (*J*_sc_), *FF*, and PCE values were obtained from the *J-V* curves. External quantum efficiency (EQE) was measured using a QE system (QE-R, Enlitech, Taiwan, China) with a chopper frequency of 210 Hz. Electrochemical impedance spectroscopy (EIS) was measured using an electrochemical workstation (Zennium Zahner CIMPS, Kronach, Germany) with a frequency from 50 KHz to 50 mHz in the dark state. The dark *J-V* and space charge limited current (SCLC) analysis were measured using a semiconductor analyzer (Keysight B1500A, Santa Rosa, CA, USA) in the dark state. During long-term shelf stability characterizations, low-bandgap PSCs were kept in a nitrogen glovebox with oxygen < 0.1 ppm and water < 0.1 ppm, and temperature around 20 °C. The PSCs were measured under AM 1.5 G 100 mW/cm^2^ illumination every 48 h.

In order to characterize the detailed performance of the as-prepared PPDs, a 450 nm pulse laser (SPUR-450 CK13212, Changchun, China) was used to emit continuous or pulsed light, and the pulse frequency can be adjusted. The waveforms of the output signals of the PPDs were examined by a oscilloscope (Tektronix MDO 3102, Biferton, Melrose, MA, USA) with an input impedance of 50 Ω [24]. The −3 dB bandwidth refers to the frequency at which the amplitude drops to 1/√2 of the maximum value. The rise (fall) time is defined to be the normalized signal varies from 10% to 90% of the peak value (from 90% to 10%). The signals observed in the oscilloscope was used to determine the bandwidth and observe the rising edge to read the rise time.

In the experiments of visible light communication for audio signal transmission, the audio signal derived a white LED through a constant current of 0.2 mA, and the single PPD without any amplifiers or filters received the optical signal and converted it into an electrical signal, which was used as the input signal of the speaker to play the audio. Moreover, PPD array was constructed by electrically connecting four PPDs in parallel, and an inductor of 100 mH was introduced in a series between the negative electrodes of two adjacent PPDs to avoid the addition of capacitors.

## 3. Results and Discussion

**Figure 1:**
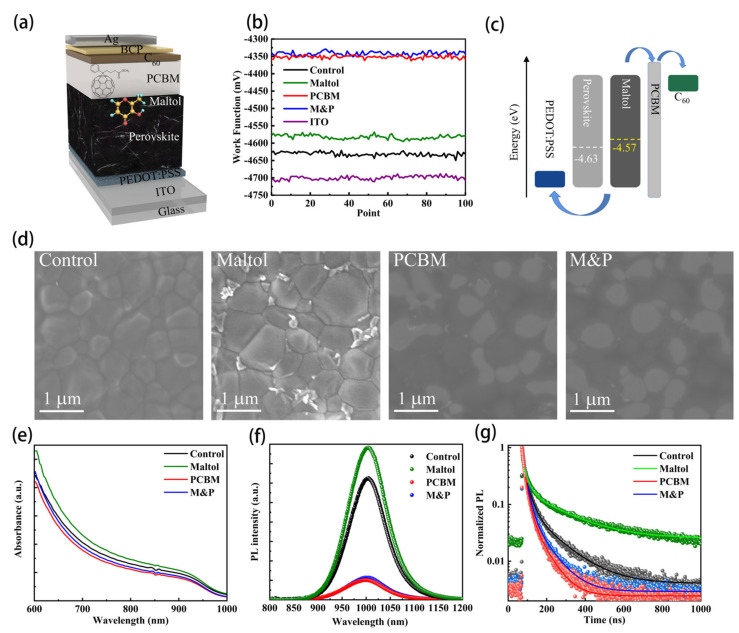
(**a**) Structure diagram of low-bandgap perovskite device with maltol and PCBM co-modification, (**b**) Surface work function profiles of the four different films, (**c**) Energy level diagrams of the maltol and PCBM co-modified device, (**d**) Top-view SEM images, (**e**) absorbance curves, (**f**) PL spectra, and (**g**) TRPL decays.

The schematic illustration of the low-bandgap perovskite devices with an inverted structure is shown in Figure 1a. Four kinds of devices were prepared to scrutinize the synergistic effects of maltol and PCBM on the optoelectronic performance: the one without any treatments (referred to as control), the device with maltol post-treatments on the perovskite film surface (referred to as maltol), the device with PCBM interlayer on the surface of perovskite film (referred to as PCBM), and the device with both maltol post-treatments and a PCBM layer (referred to as M&P).

Surface work functions of perovskite films with different treatments are shown in Figure 1b. The control perovskite film has a Fermi level of −4.63 eV, whereas after maltol post-treatment, the Fermi energy level of perovskite layer upshifted to −4.58 eV, which should be related to the alleviated p-type self-doping due to the suppressed Sn^2+^ oxidization [25]. Moreover, the PCBM-coated perovskite film has a surface work function of −4.35 eV, which should be ascribed to the intrinsic high work function of PCBM. Therefore, the introduction of PCBM forms a double layer electron transporting configuration with the following evaporated C_60_ layer, thus improving the built-in electric field compared to the control device. However, for the device with both maltol and PCBM treatments, the work function level is determined to be −4.34 eV, which is almost the same as solely PCBM-based samples. We drew a scheme picture of the energy level alignment of the low-bandgap perovskite devices as Figure 1c, where the maltol-modified absorber layer has a slightly upshifted Fermi level, whereas the PCBM interlayer introduces a small energy spike at the back intersurface [26].

Surface morphologies of perovskite films are shown in Figure 1d. The control perovskite film has a homogenous surface with the averaged grain size of ~700 nm. After maltol post-treatment, the film surface shows more clear grains, and the grain boundaries (GBs) are filled with bright nanoparticles, which is ascribed to the formation of metal halides [27]. The PCBM-coated samples (solely PCBM and M&P) show relatively blurred surfaces due to the thick layer of organic materials. The corresponding surface roughness of the four samples were further characterized using atomic force microscope (AFM), and the results are shown in Figure S3. The root mean square (RMS) roughness are obtained to be 40.6, 24.6, 39.6, and 20.3 nm, respectively, for the control, maltol, PCBM, and M&P samples, and the detailed information is summarized in Table S1. The control perovskite film has the highest roughness of 40.6 nm, whereas after maltol post-treatment, the surface roughness reduced slightly to 39.6 nm, indicating the polishing effect of maltol solution washing. The smoother surface has the improved contact quality with the following charge transporting layers and the better carrier extraction capability [28].

The maltol post-treatment substantially enhances light absorption due to the removing of defects [19], whereas the introduction of PCBM slightly decreases the absorption which might be caused by the mismatched refractive index between the PCBM and perovskite layers [29]. The trap density and passivating capability of the maltol and PCBM on the low-bandgap perovskite films were further explored by the PL and TRPL characterizations. Figure 1f shows that the perovskite film upon maltol post-treatment has improved PL intensity compared to the control sample, indicating the positive effect on reducing surface trap and suppressed charge recombination. However, after PCBM coating, the PL intensity of perovskite film decreases significantly due to the fast charge extraction. The perovskite film with both maltol and PCBM has the lowest PL intensity, implying high film quality with fast charge transportation and extraction. Based on the TRPL results in Figure 1g, the perovskite film with maltol treatment has the longer carrier lifetime (i.e., 48 ns) compared to the control one (i.e., 38 ns), which further confirms the reduced trap density. Meanwhile, the perovskite film with PCBM coating has significantly shorter carrier lifetime (i.e., 18 ns) than the M&P co-modified perovskite film (i.e., 22 ns), indicating the improved carrier transfer from the film bulk to the PCBM layer. Therefore, after combining maltol and PCBM treatments, the perovskite films have significantly reduced defects, leading to enhanced crystallinity and better charge transfer dynamics.

**Figure 2:**
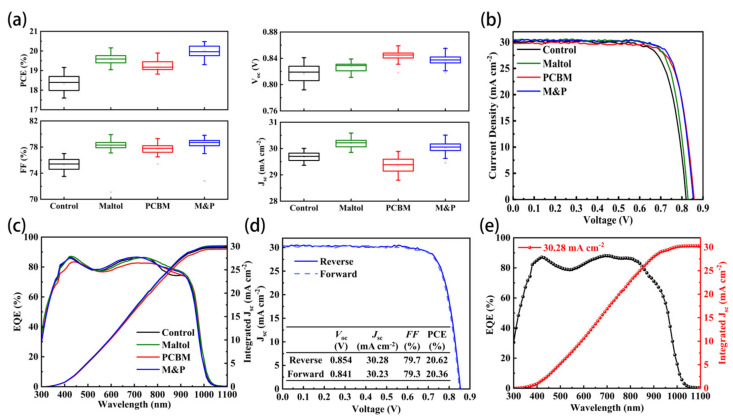
Photovoltaic performance of the four kinds of PSCs. (**a**) Statistical results of *V*_oc_, *J*_sc_, *FF* and PCE of the four different PSCs, (**b**) *J-V* curves under reverse scan and (**c**) EQE plots of PSCs with different treatments, (**d**) *J-V* curves of the champion device under reverse and forward scan, and (**e**) EQE curve of the champion device.

To further confirm the positive effects of our treatments, we fabricated a large number of low-bandgap PSCs. The statistical results of photovoltaic performance of the four kinds of PSCs, including control, maltol-based, PCBM-coated, as well as maltol and PCBM co-modified devices, are shown in Figure 2a. The *V*_oc_ has been improved with various treatments. Maltol decoration slightly enhances the *V*_oc_ from 0.817 V to 0.827 V due to the reduced trap densities, and PCBM introduction significantly increases the *V*_oc_ to 0.844 V due to the elevated lowest unoccupied molecule orbital (LUMO) energy. Although PCBM treatment has positive effects on *V*_oc_, it severely compromises the *J*_sc_, which is related to the large charge transfer barrier at the perovskite/ETL interface. Moreover, maltol-based PSCs have highly increased *J*_sc_ results, whereas the M&P co-modified PSCs have slightly lower *J*_sc_ than the solely maltol-treated ones. The averaged *J*_sc_ of control, maltol-based, PCBM-based, and M&P co-modified PSCs are 29.7, 30.2, 29.4, and 30.0 mA cm^−2^, respectively. In addition, devices with different treatments have a highly improved fill factor (*FF*) and power conversion efficiency (PCE) compared with the control devices. The averaged PCE of the control devices is about 18.5%, whereas the PCE increases to 19.5% and 19.2% after maltol passivation and PCBM coating, respectively. Finally, the PSCs with both maltol and PCBM have the best performance with an averaged PCE of larger than 20%. Since maltol has a positive effect on optical and charge transfer properties, we further studied the effect of maltol concentration on the device performance in the presence of PCBM. Finally, the concentration was optimized to be 1 mg/mL (Figure S5).

Figure 2b shows the representative *J-V* curves of the four kinds of low-bandgap PSCs. The maltol-based PSC has highly enhanced *J*_sc_ and *FF*, and slightly increased *V*_oc_, whereas the PSCs with PCBM layers, including solely PCBM-based and M&P co-modified, have significantly improved *V*_oc_. The optimal condition was found to be the presence of both maltol and PCBM, with the advantages of high *V*_oc_, *J*_sc_, and *FF*. The detailed photovoltaic parameters under various conditions are listed in Table 1. The external quantum efficiency (EQE) plots of the four PSCs are shown in Figure 2c. The maltol-based PSC has the best EQE response, especially at the long-wavelength region, which should be attributed to the highly improved film quality with fewer defects. However, PCBM introduction sacrifices the EQE response due to the parasitic absorption of PCBM at the rear interface with fewer photons reflected back (Figure S7). However, for the M&P co-modified PSCs, the positive effect of maltol could offset the current loss by PCBM, thus giving reasonable EQE amplitude. Figure 2d shows the *J-V* curves of the champion device under both forward and reverse scans. The PCE was obtained to be 20.62 (20.36)% under a reverse (forward) voltage scan, with *V*_oc_ of 0.854 (0.841) V, a *J*_sc_ of 30.28 (30.23) mA cm^−2^, and a *FF* of 79.7 (79.3)%. It shows that our prepared low-bandgap PSCs have negligible hysteresis. The corresponding EQE spectrum is shown in Figure 2e, with the integrated *J*_sc_ of 30.28 mA cm^−2^.

**Figure 3:**
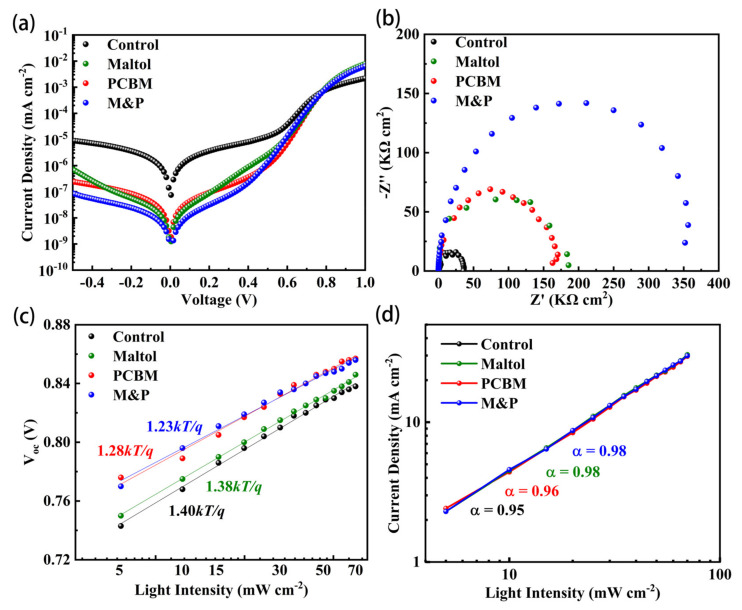
Characterizations of electrical properties of the four different PSCs. (**a**) Dark *J-V* curves, (**b**) Nyquist plots, (**c**) *V*_oc_, and (**d**) *J*_sc_ as a function of light intensity.

We further studied the underlying mechanisms of the improved device performance from the maltol and PCBM treatments. The dark current density was analyzed first since it is closely related to the possible current leakage in PSCs [30]. Figure 3a compares the dark current densities of the four kinds of devices. The control one has the highest saturated current, indicating severe charge recombination, whereas with maltol passivation, the saturated current reduced about two orders of magnitude, confirming the suppressed current leakage due to the reduced defects. Moreover, using a PCBM interlayer shows similar results, i.e., current loss is highly eliminated due to the increased built-in electric field and charge barrier. The co-modified PSCs have the lowest saturated current, taking advantage of both maltol and PCBM.

Electrochemical impedance spectrum (EIS) was measured to reveal the possible charge transfer dynamics. Figure 3b displayed the Nyquist plots of the four different PSCs. Generally, a Nyquist plot has a small semicircle at high frequency region, and a large semicircle at low frequency region, corresponding to charge transfer resistance and recombination resistance, respectively. As shown in Figure 3b, all the presented PSCs have a clear big semicircle, whereas the small semicircle is hardly detected. The control device has the smallest semiarch, indicating the worst recombination resistance, consistent with the highest risk of charge recombination due to the worst film quality and severe defects. Devices with either maltol or PCBM treatment have a dramatically enlarged radius of the semicircle, indicating the larger equivalent recombination resistance due to the positive passivating, whereas the maltol and PCBM co-modified PSC shows the largest semiarch, implying the maximized recombination resistance with the lowest carrier recombination. In the start points of the Nyquist plots (Figure S8), the relatively larger value of the PCBM-based PSC further confirms the energy barrier due to the upshifted energy level of PCBM at the rear interface.

We further discussed carrier recombination by measuring the light intensity dependence of *V*_oc_ and *J*_sc_. The *V*_oc_ follows a liner relationship versus the logarithmic light intensity according to $V_{\mathrm{oc}}=\left(\frac{nk_{B}T}{q}\right)\ln\left(\frac{I}{I_{0}}+1\right)$, where *k*_B_ is the Boltzmann constant, *T* is the temperature, *q* is an elementary charge, and *n* is the ideal factor. Generally, a smaller ideal factor close to 1 means a lower Shockley-Read-Hall (SRH) recombination level in a photovoltaic device. As shown in Figure 3c, control, maltol-based, PCBM-based, and M&P co-modified PSCs have the *n* values of 1.40, 1.38, 1.28, and 1.23, respectively, indicating the lowest SRH recombination in the PSC with both maltol and PCBM treatments. We further evaluated the trap density of control perovskite and maltol-based perovskite films by SCLC analysis (Figure S9). The trap density of the maltol-based device is 1.1 × 10^15^ cm^−3^, which is lower than that of control device (1.7 × 10^15^ cm^−3^). These results are consistent with the saturate current and EIS analysis. Figure 3d provides the *J*_sc_ evolutions as a function of light intensity. *J*_sc_ has a linear relation with light intensity (*P*), according to *J*_sc_∝*P*^α^. As shown in Figure 3d, all the PSCs have a similar slope approaching 1, indicating the small space charge limited current [31,32]. However, the relatively smaller α values in the PCBM-presented PSCs indicate a slight charge barrier in the device. We provided the long-term shelf stability of PSCs with different treatments, as shown in Figure S10. The M&P co-modified device maintained more than 90% of its initial PCE after 720 h storage, whereas the control one kept less than 70% of its original efficiency. The improved stability of the optimized device should be ascribed to the synergistic effects of maltol and PCBM at the back interface of perovskite/ETL with fewer defects and ion migrations.

Considering that the PSCs can also work well as self-powered PPDs, we then demonstrate that our prepared low-bandgap PSCs can be directly used as a signal receiver without any amplifiers or filters in a visible light communication (VLC) system [33,34]. VLC technology use a transmitter to send out high speed light flashing signals for transmit information, and the visible lights usually fall into the wavelength region of 380–750 nm [35,36]. Visible light communication is safe and compatible to daily light illumination without additional electromagnetic radiation. Light emitting diodes (LEDs) with white color are commonly used as the light source and transmitter due to their low power consumption, high luminous power, and low-cost benefits [37,38,39]. The detector at the receiving of visible light communication generally requires an additional power supply, which complicates the visible light communication system, but self-powered PPDs do not have this problem and can work without a power supply. At the same time, self-powered PPDs have satisfactory performance, including fast response speed, high responsivity, and so on.

**Figure 4:**
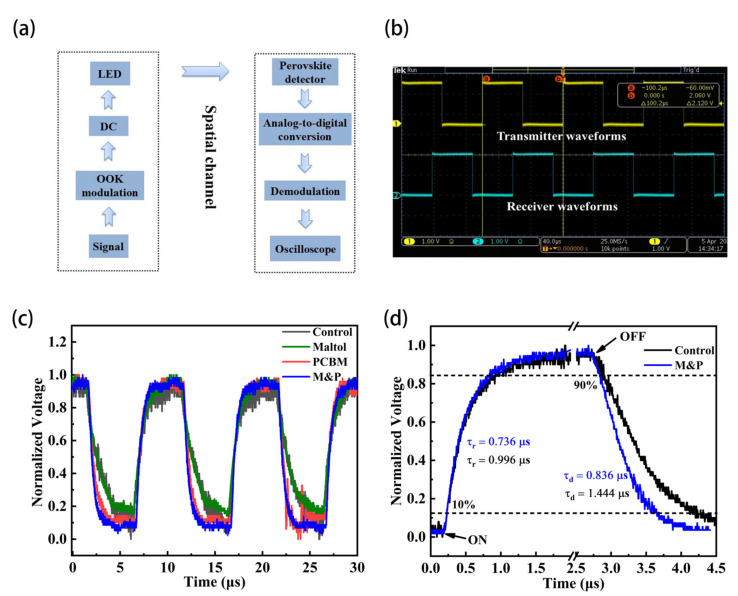
(**a**) Flow chart of visible light communication, (**b**) Transmitter waveforms and receiver waveforms in visible light communication system, (**c**) The digital data waveforms of the devices with four different treatments (Control, Maltol, PCBM and M&P), and (**d**) Transient light response of the devices with two different treatments (Control and M&P).

Figure 4a shows the scheme of typical working process of a VLC system. The digital signal is first modulated by on off keying (OOK) protocol [40]. In our experiments, field programmable gate array (FPGA, model EP4CE10) was used to do the modulation, demodulation, and conversion processes. One magnitude is taken as zero, the other is taken as non-zero. The constant bias was used to lighten the LEDs on, and an additional programed data was applied on the driven voltage for flashing illumination, and our broadband self-powered PPDs are employed as the signal receivers that convert the irradiated light to photocurrent, and then give the data output. The photocurrent is first converted from an analog signal to a digital signal. Then, the digital signal can be demodulated. Finally, we can observe the waveforms from an oscilloscope, and the transmitted audio signals can also be played through a speaker.

Figure 4b shows that there is no distortion in both the input and output signals for the 100 KHz square waves. The observed signal obtained by the self-powered PPD is represented by the cyan color, which matches well with the original input signal shown in yellow color. The constant and tiny delay between the transmitter waveforms and receiver waveforms is decided by the program code, and could be tuned by different protocols.

Figure 4c compares the responses of the four different PPDs to the same input square signal. We can see that all the PPDs have similar response shapes; however, the rising and falling edges are different. In particular, we calculated the response time of the control and optimized target devices, relying on the photocurrents of the devices with at a pulse frequency of 100 kHz at 0 bias (Figure 4d). The control and target devices have the rise time of 0.996 and 0.736 μs, respectively, and the corresponding decay time is calculated to be 1.444 and 0.835 μs. The target PPD with both maltol and PCBM treatments is faster compared to the control, which should be related to the better film quality and charge transfer properties. The rise time and fall time for the four different devices are shown in Figure S11a–d.

**Figure 5:**
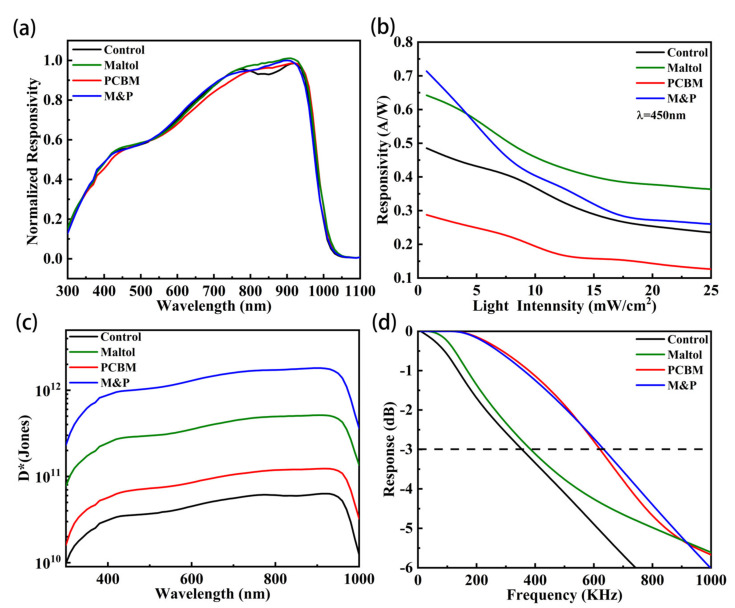
Photodetection performance of the four different perovskite photodetectors. (**a**) Responsivity spectra, (**b**) Light intensity dependent responsivity, (**c**) Specific detectivity, and (**d**) Frequency response.

The responsivity spectra as a function of wavelength for control and target PPDs are shown in Figure S12a. The responsivity is determined by the *EQE* and the wavelength as follows: [41]

(1) $$R=\frac{EQE\;\cdot \;\lambda }{hc}$$

where *h* is Planck constant, *c* is the speed of light, and *λ* is the wavelength. The normalized responsivity spectra of the four different devices are shown in Figure 5a. Compared with the control one, the target PPD has an increased *EQE* and photocurrent and, thus, improved responsivity.

Figure 5b shows the responsivity (*R*) as a function of light intensity for the four different PPDs. Maltol and PCBM treatments could suppress Sn oxidization and reduce the defect density, thus being responsible for the increased responsivity. The target PPD with both maltol and PCBM shows obviously greater responsivity than the control (Figure S12b). The responsivity for all the devices decreases as light intensity increases. At low light intensities, the strong built-in electric field reduces the recombination rate resulting in high responsivity. However, at higher light intensities, the recombination rate increases due to the reduced built-in-potential reducing the responsivity [42].

The specific detectivity (D*) indicates detection capability of weak light [43], and is determined by the dark current, responsivity, and bandwidth. Higher values of D* enable better detection of fainter light. D* can be expressed by the following equation: [41]

(2) $$D^{*}=\frac{\sqrt{AB}\;\cdot \;R}{I_{dark}}$$

where *A* is the devices working area, *R* the optical responsivity, *B* the bandwidth, and *I_dark_* the dark current. Figure 5c shows the D* spectra of the four different PPDs. The peak D* of about 1.814 × 10^12^ is obtained at around 900 nm wavelength for the devices with M&P. Maltol and PCBM could sufficiently passivate defects and tailor energy level arrangement, and therefore lead to high-performance PPDs with the improved responsivity and reduced dark current.

The frequency response to modulated light is shown in Figure 5d. The −3 dB cut-off frequency of the target PPD is 636 KHz, higher than the others. Table S2 shows rise times, decay times, and bandwidth of four PPDs, and the device with both maltol and PCBM shows the best performance in our work. Figure S12c further indicates that the target PPD with M&P has a more stable performance than the control device, which should be ascribed to the better film quality and being less vulnerable to additional stresses. Moreover, we summarized the key performance parameters of perovskite detectors in Table S3 and found that our low-bandgap broad-band devices are in the first class among the self-powered PPDs.

Finally, the optimized low-bandgap PSCs are demonstrated to be used as the self-powered broadband PPDs for the receiver without any amplifiers or filters in a visible light communication system. We provided Video S1 that shows the successful application of our self-powered PPDs acting as the signal receiver in a VLC system. When the distance between the LED and the perovskite detector increases, the received signal is weakened. When the light is blocked, the signal cannot pass, and the audio signal cannot be played. In order to increase the transmission distance, two or more detectors can be connected in parallel. However, the direct parallel connection reduces the −3dB bandwidth. Thus, we made the PPDs array so that four detectors were electrically connected in parallel with additional inductors. A comparison among a single perovskite detector, four perovskite detectors in parallel, and four perovskite detectors in parallel with inductors are shown in Table S4, which implies that our prepared self-powered PPDs have large development potential in the future for VLC due to their fast photoelectric conversion and good detection ability.

Currently, we present a proof-of-concept fabrication strategy for the low-bandgap perovskite devices in lab scales. More efforts should be devoted to the ambient stability and large-scale fabrication issues of Sn-based perovskites to improve the possible industrial applications.

## 4. Conclusions

In conclusion, we demonstrate the synergistic effect of maltol and PCBM on improving the film quality and device performance of low-bandgap perovskites. Maltol post-treatment could sufficiently passivate the absorber layer with suppressed Sn^2+^ oxidization and Sn vacancies formation. A PCBM interlayer upshifted the built-in electric field and reduced halide distortion and ion migration, thus enhancing the *V*_oc_ and performance of the PSCs. The best PCE of low-bandgap PSCs was obtained to be 20.63% with negligible hysteresis. Moreover, the optimized PSCs are further applied as self-powered PPDs, and employed as the signal receiver in a VLC system. The single unbiased PPD shows a low saturated current of 7.31 × 10^−11^ A, a fast response speed of 0.736 μs, and the responsivity of 0.554 A/W. We also propose a successful application of the as-prepared low-bandgap PSCs as the broadband self-powered PPDs upon an audio transmission.

## Figures and Tables

**Table 1:** A comparison of the photovoltaic performance parameters based on the four treatments (Control, PCBM, Maltol, and M&P) under reverse scan.

	*V*_oc_ (V)	*J*_sc_ (mA cm^−2^)	*FF* (%)	PCE (%)
Control	0.821	30.08	76.8	18.96
Maltol	0.829	30.48	79.1	19.98
PCBM	0.858	29.80	78.1	20.00
M&P	0.854	30.28	79.7	20.62

## Data Availability

The data presented in this study are available on request from the corresponding author.

## Supplementary Materials

The following supporting information can be downloaded at: https://www.mdpi.com/article/10.3390/nano12122065/s1, Figure S1: Photovoltaic parameters of the perovskite solar cells based on different concentrations of PCBM solution. Figure S2: EQE curves of the perovskite solar cells based on different concentrations of PCBM solution. Figure S3: AFM images of the perovskite surfaces based on the four different structures. Figure S4: EQE curves of the perovskite solar cells with various maltol concentrations. Figure S5: Statistics of photovoltaic performance of PSCs with various maltol concentrations. Figure S6: SEM images of the different film surfaces. Figure S7: Optical bandgap, absorptivity curves, and reflectance & transmittance curves of the four different structures. Figure S8. Nyquist plots of the four different PSCs under high frequency in the start stage of measurement. Figure S9: SCLC measurement of the control and target devices with hole-only structure. Figure S10. Device shelf stability of unencapsulated PSCs kept in glovebox with oxygen and water less than 0.1 ppm for different time. Figure S11: Transient light response of the four different perovskite photodetectors. Figure S12: Light intensity dependent responsivity, normalized responsivity spectra, and stability test of the normalized photocurrent response. Table S1: Surface roughness of the four different structures. Table S2: Rise time, decay time, and bandwidth of the four different perovskite photodetectors. Table S3: A performance comparison between the reported perovskite photodetectors and this work. Table S4: A comparison of a single perovskite detector and four perovskite detectors in parallel without or with inductors; Video S1: Audio transmission demonstration. References [24,42,44,45,46,47,48,49,50,51] are cited in the Supplementary Materials.
