# Supplementary material for: Back Interface Passivation for Efficient Low-Bandgap Perovskite Solar Cells and Photodetectors
Source: Nanomaterials (Basel). 2022 Jun 15;12(12):2065. doi: 10.3390/nano12122065 (PMC9231224; doi:10.3390/nano12122065)
Supplement: Supplementary file 1 [file nanomaterials-12-02065-s001.zip › nanomaterials-1752031-supplementary/Supplementary File/Supplementary information-revised.pdf]

Supplementary information

Article

Back Interface Passivation for Efficient Low-Bandgap Perovskite Solar Cells and Photodetectors

Jiayu Lu [1,†], Huayang Wang [1,†], Tingbing Fan [1], Dong Ma [2,*], Changlei Wang [1,*], Shaolong Wu [1,*] and Xiaofeng Li [1]

1 Collaborative Innovation Center of Suzhou Nano Science and Technology, Key Lab of Advanced Optical Manufacturing Technologies of Jiangsu Province & Key Lab of Modern Optical Technologies of Education Ministry of China, School of Optoelectronic Science and Engineering, Soochow University, Suzhou 215006, China; 20205239017@stu.suda.edu.cn (J.L.); 20204239018@stu.suda.edu.cn (H.W.); 20204239018@stu.suda.edu.cn (T.F.); xfli@suda.edu.cn (X.L.)

2 School of Rail Transportation, Soochow University, Suzhou 215137, China

* Correspondence: madong@suda.edu.cn (D.M.); cl.wang@suda.edu.cn (C.W.); shaolong_wu@suda.edu.cn (S.W.)

† These authors contributed equally to this work.

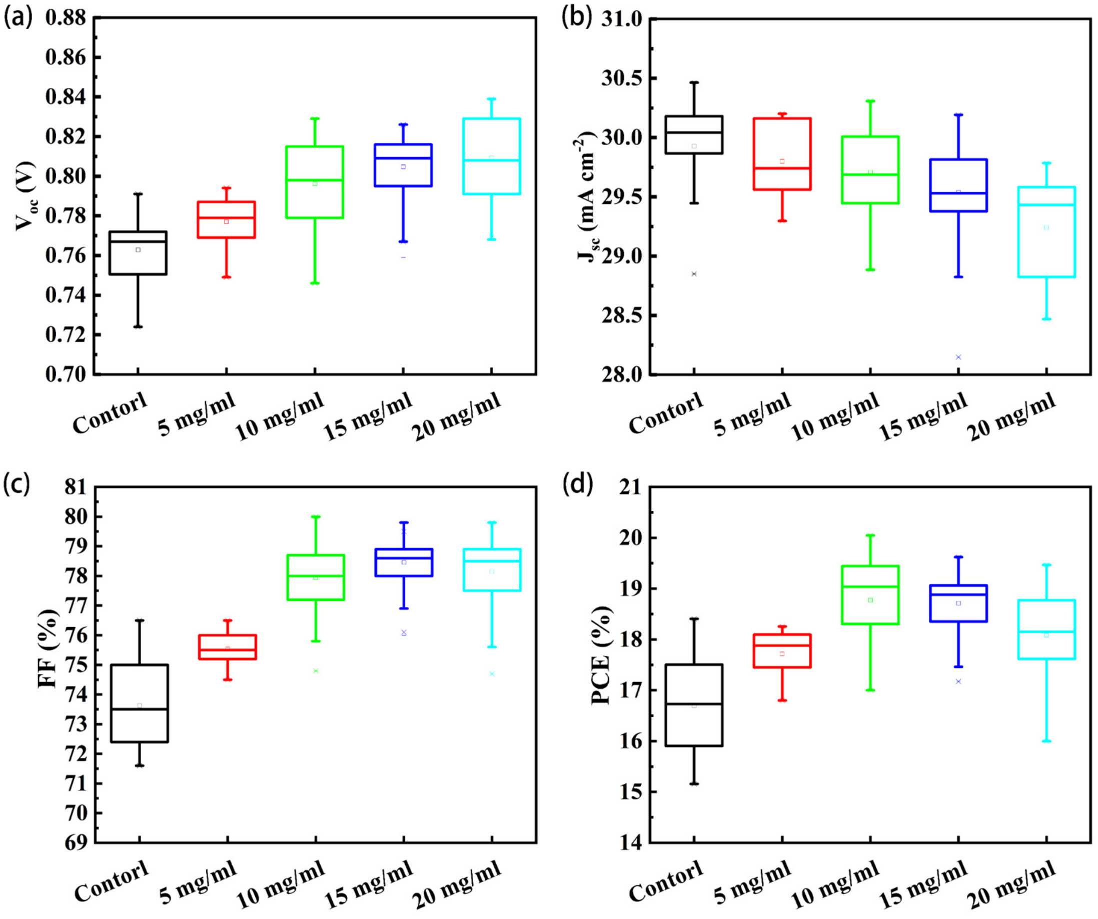

Figure S1. Photovoltaic parameters of the perovskite solar cells based on different concentrations of PCBM solution including control (0 mg/mL), 5 mg/mL, 10 mg/mL and 20 mg/mL. (**a**-**d**) V_{oc}, J_{sc}, *FF*, and

PCE statistical chart for the devices with the structure of glass/ITO/PEDOT:PSS/Perovskite/PCBM /C_{60}/BCP/Ag.

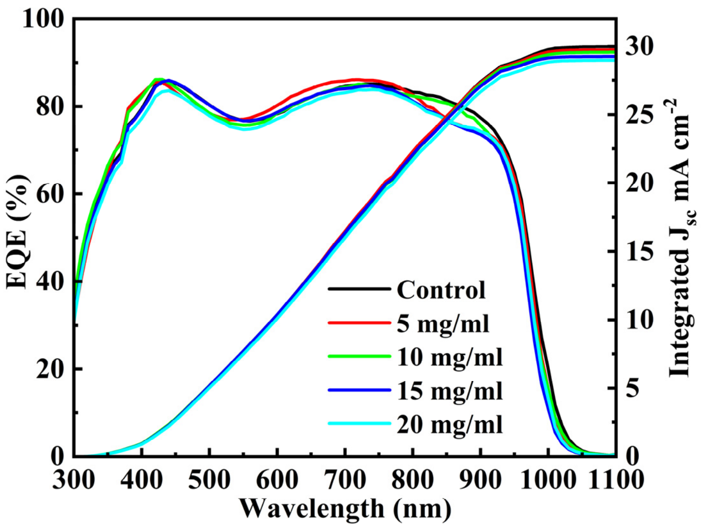

Figure S2. EQE curves of the perovskite solar cells based on different concentrations of PCBM solution. With an increase of concentration, the integrated current density is 29.98 mA cm^{-2}, 29.74 mA cm^{-2}, 29.57 mA cm^{-2}, 29.25 mA cm^{-2} and 28.97 mA cm^{-2}, respectively.

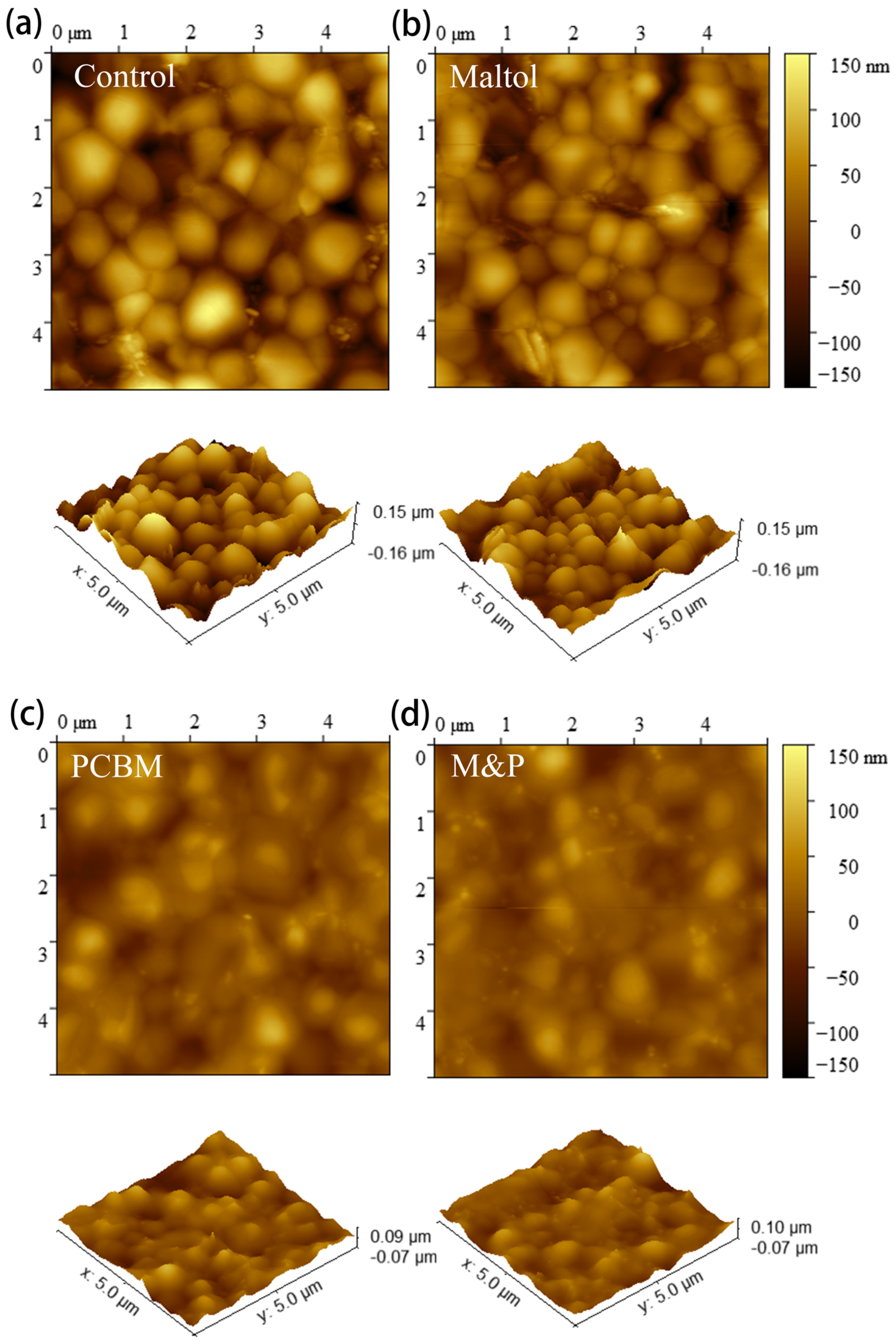

Figure S3. AFM images of the perovskite surfaces based on the four different structures involving (**a**) Control (Glass/ITO /PEDOT/Perovskite), (**b**) Maltol (Glass/ITO/PEDOT:PSS/Perovskite/Maltol), (**c**) PCBM (Glass/ITO/PEDOT:PSS /Perovskite/PCBM), (**d**) M&P (Glass/ITO/PEDOT:PSS/Perovskite/ Maltol/PCBM).

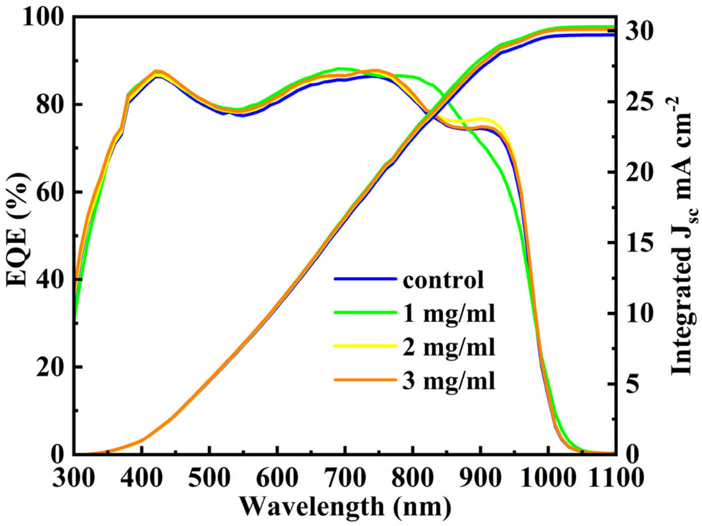

Figure S4. EQE curves of the perovskite solar cells with various maltol concentrations (0 mg/mL, 1 mg/mL, 2 mg/mL, 3 mg/mL), the integrated current density is respectively 29.72 mA cm^{-2}, 30.28 mA cm^{-2}, 30.11 mA cm^{-2}, 30.12 mA cm^{-2} with the increasing Maltol concentration.

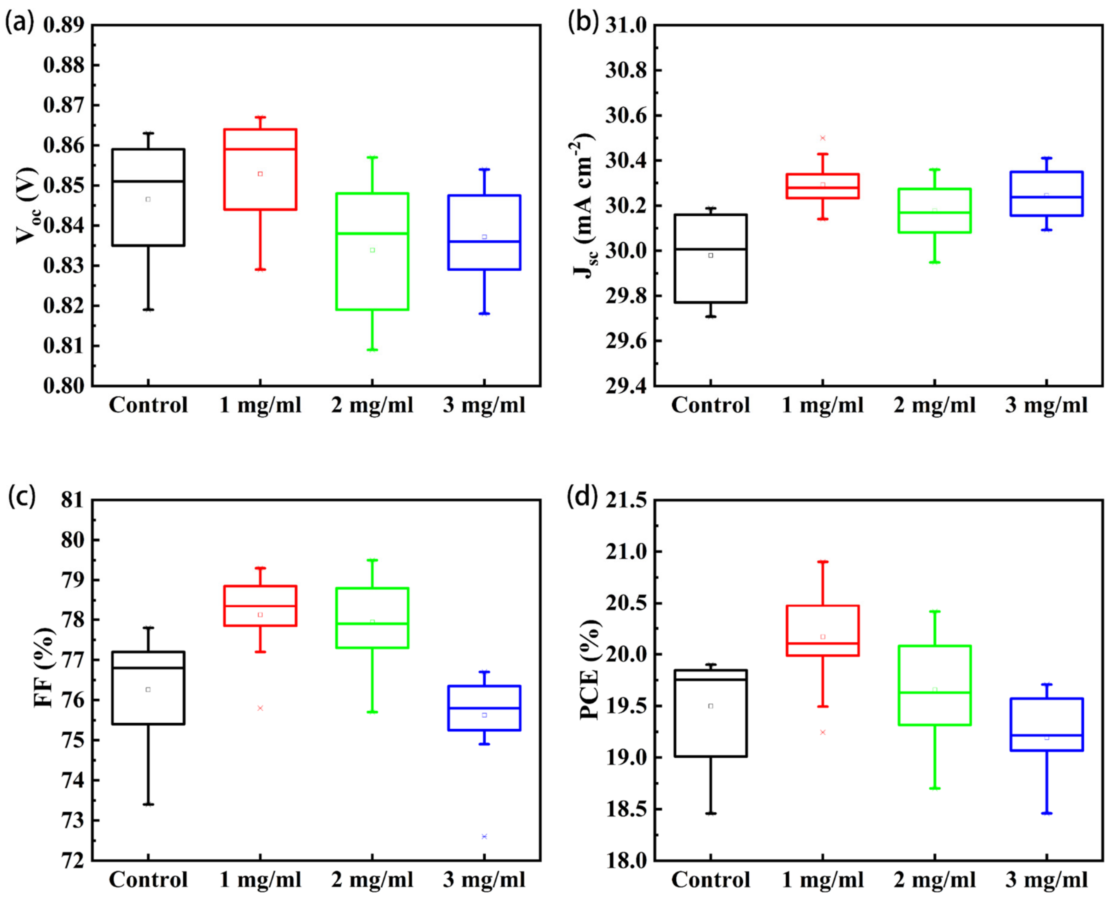

Figure S5. Photovoltaic performance of perovskite solar cells with various maltol concentrations involving control (0mg/mL), 1 mg/mL, 2 mg/mL, and 3 mg/mL, based on the device structure of Glass/ITO/PEDOT:PSS/Perovskite/Maltol/PCBM/C60/BCP/Ag. (**a**-**d**) V_{oc}, J_{sc}, *FF*, and PCE, respectively.

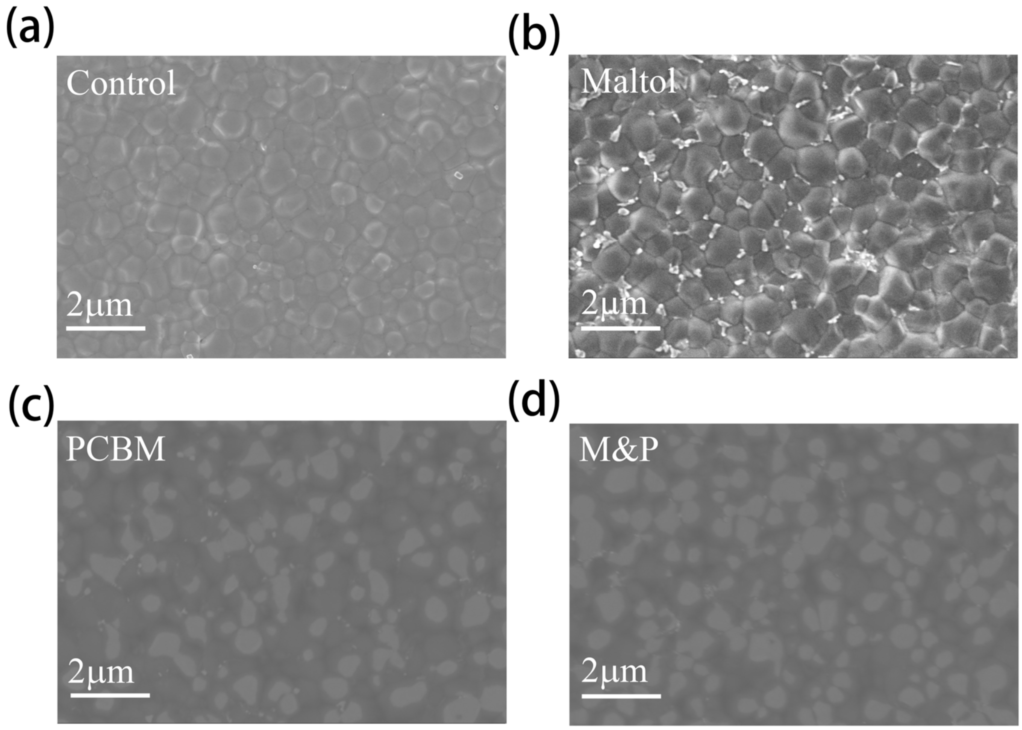

Figure S6. SEM images of the different device surfaces with (**a**) control, (**b**) maltol, (**c**) PCBM, and (**d**) M&P.

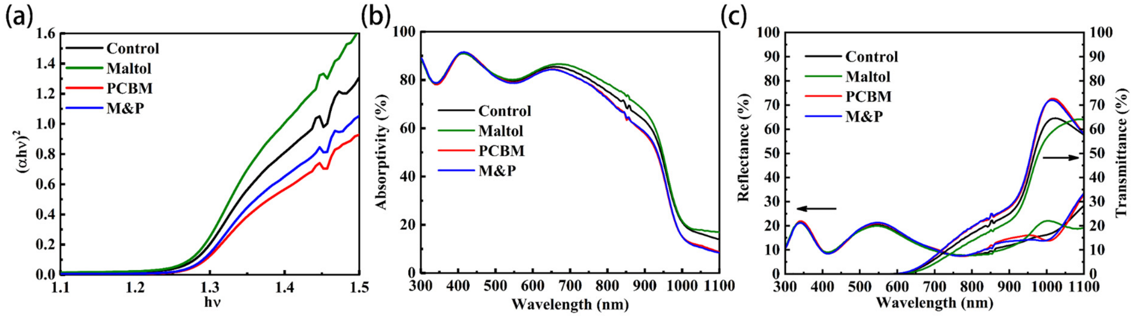

Figure S7. (**a**) Optical bandgap, (**b**) absorptivity curves, and (**c**) reflectance & transmittance curves of the four different structures, including Glass/ITO/PEDOT/Perovskite (Control), Glass/ITO/PEDOT/Perovskite/PCBM (PCBM), Glass/ITO/PEDOT/Perovskite/Maltol (Maltol) and Glass/ITO/PEDOT/Perovskite/Maltol/ PCBM (M&P). The bandgap of Perovskite is between 1.25 eV and 1.26 eV uninfected by the structure variation.

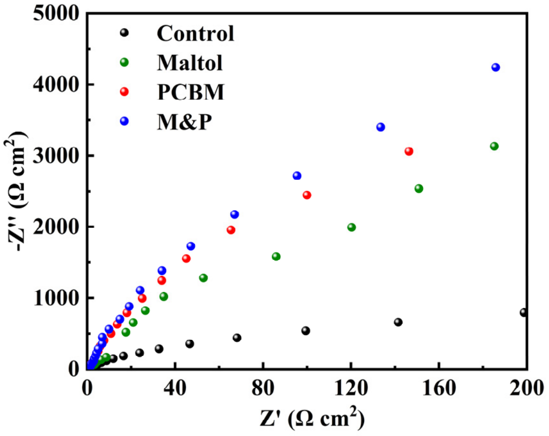

Figure S8. Nyquist plots of the four different PSCs under high frequency in the start stage of measurement.

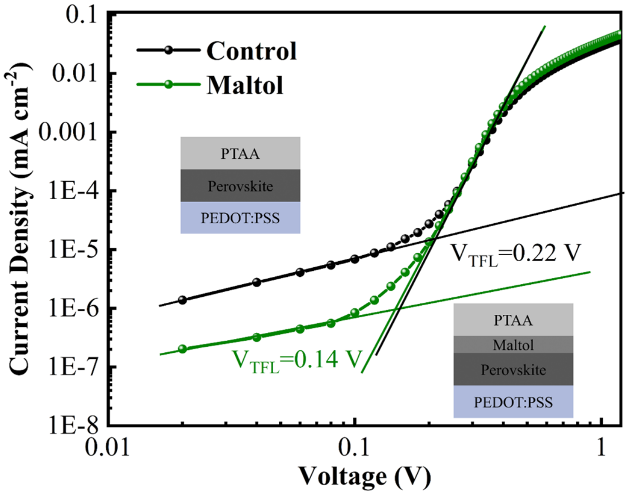

Figure S9. SCLC measurement of the control and target devices with hole-only device structure as shown in the inset (Glass/ITO/PEDOT:PSS/Perovskite/PTAA/Cu and Glass/ITO/PEDOT:PSS/ Perovskite/Maltol/PTAA/ Cu). Maltol is spin coated between perovskite and PTAA layers. The trap-filled limit voltage (V_{TFL}) of control device and maltol-based device are 0.22 V and 0.14 V, respectively.

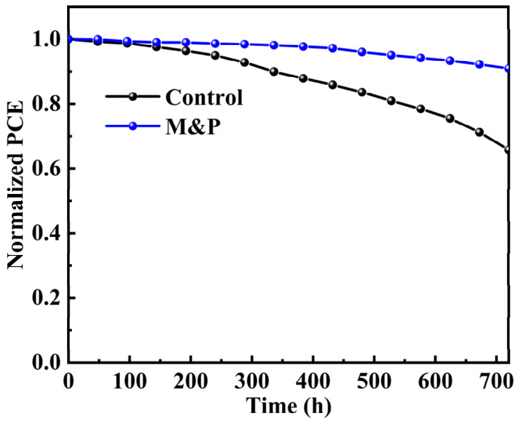

Figure S10. PCE evolution of the unencapsulated control and M&P devices in glovebox for different time.

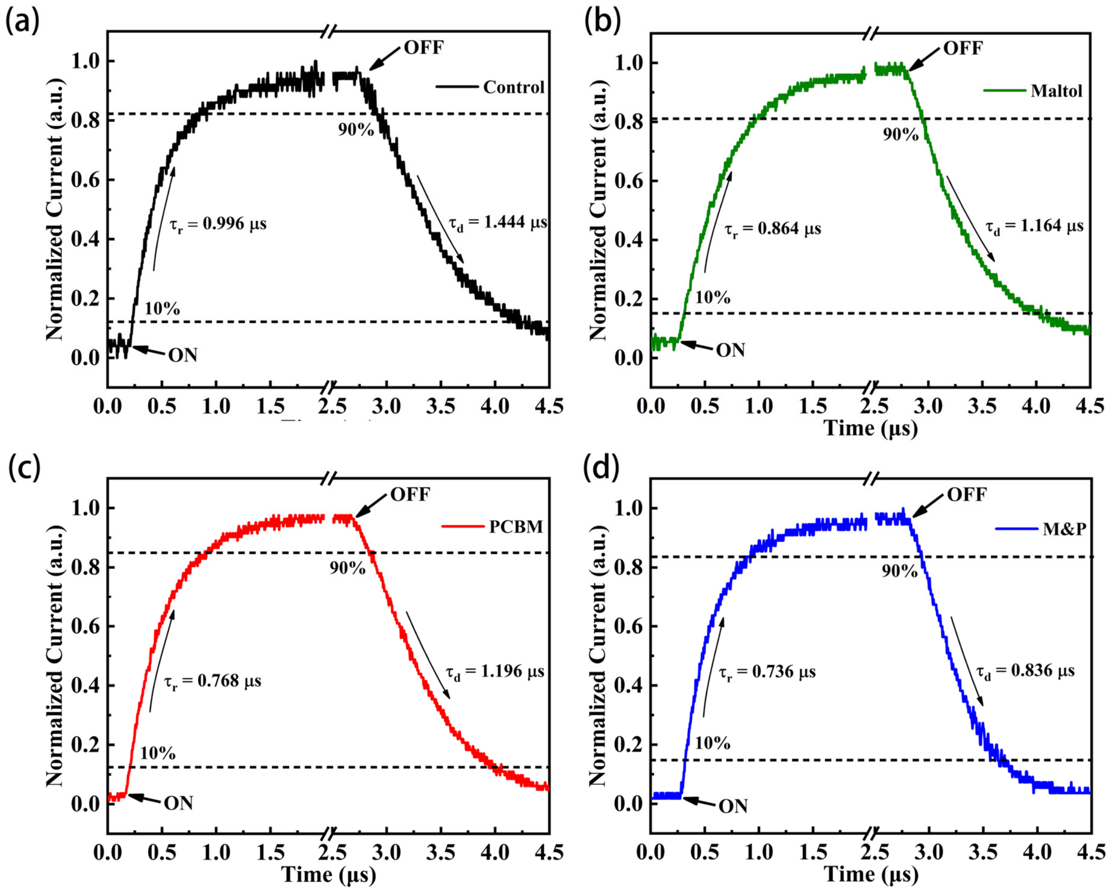

Figure S11. (**a-d**) Transient light response of the four different perovskite photodetectors.

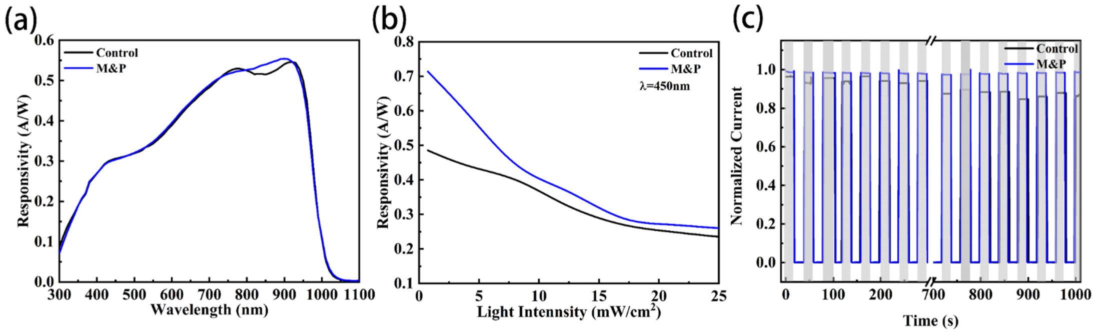

Figure S12. (**a**) Light intensity dependent responsivity. (**b**) normalized responsivity spectra. (**c**) Stability test of the normalized photocurrent response.

Table S1. Surface roughness of the four different structures (Control, PCBM, Maltol and M&P).

Roughness (nm)	Control	Maltol	PCBM	M&P
R_q	40.6	39.6	24.6	20.3
R_a	32.6	30.7	19.4	15.3

Table S2. Rise time, decay time, and bandwidth of the four different perovskite photodetectors.

Structure	τ_r(μs)	τ_d(μs)	bandwidth (KHz)
Control	0.996	1.444	353
Maltol	0.864	1.164	363
PCBM	0.768	1.196	625
M&P	0.736	0.836	636

Table S3. A performance comparison between the reported perovskite photodetectors and this work.

Description	response time	responsivity (A/W)	bias(V)	I_{dark} (A)	references
$MAPbI_{3-x}Cl_x$	160 ns		0		[1]
$MAPbI_3$	120 ns		0		[2]
$MAPbI_3$	25.8 / 0.62 ms		0.1		[3]
$MAPbI_3$	12.7 /6.9 μs	0.0637	0		[4]
$MAPbI_3$	13.8 /16.1 μs	0.1611	0	2×10^{-14}	[5]
$MAPbBr_3$	70 /150 μs	0.002	0		[6]
$MAPbI_3$	460 / 940 ns	0.55	0		[7]
$MAPbI_3$	3 / 2.2 μs		0		[8]
$MAPbI_3$	1.2/3.2 μs	0.395	0		[9]
$MAPbI_3$	4.0 / 3.3 μs	0.314	0		[10]
$(FASnI_3)_{0.6}(MAPbI_3)_{0.37}(MAPbBr)_{0.03}$	0.736 / 0.836 μs	0.55428	0	7.31×10^{-11}	This work

Table S4. A comparison of a single perovskite detector, four perovskite detectors in parallel and four perovskite detectors in parallel with inductors.

Description	Response time (μs)	Bandwidth (KHz)	Photocurrent (μA)
a single perovskite detector	0.74	636	18.30
four perovskite detectors in parallel	2.80	158	73.34
four perovskite detectors in parallel with inductors	0.74	598	69.87

Supplementary References

1. Dou, L.; Yang, Y.M.; You, J.; Hong, Z.; Chang, W.H.; Li, G.; Yang, Y. Solution-processed hybrid perovskite photodetectors with high detectivity. *Nat.Commun.* **2014**, *5*, 1-6.
2. Fang, Y. Huang, J. Resolving weak light of sub-picowatt per square centimeter by hybrid perovskite photodetectors enabled by noise reduction. *Adv. Mater*. **2015**, *27*, 2804-2810.
3. Pang, T.; Jia, R.; Wang, Y.; Sun, K.; Hu, Z.; Zhu, Y.; Luan, S.; Zhang, Y. Self-powered behavior based on the light-induced self-poling effect in perovskite-based transport layer-free photodetectors. *J. Mater. Chem. C* **2019**, 7, 609-616.
4. Wang, J.; Xiao, S.; Qian, W.; Zhang, K.; Yu, J.; Xu, X.; Wang, G.; Zheng, S.; Yang, S. Self-driven perovskite narrowband photodetectors with tunable spectral responses. *Adv. Mater. 2021*, 33, 2005557.
5. Wu, C.Y.; Peng, W.; Fang, T.; Wang, B.; Xie, C.; Wang, Li.; Yang, W.H.; Luo, L.B. Asymmetric contact-induced self-driven perovskite-microwire-array photodetectors. *Adv. Electron. Mater.* **2019**, *5*, 1900135.
6. Shaikh, P.A.; Shi, D.; Retamal J. R. D.; Sheikh, A.D.; Haque, M.A.; Kang, C.F.; He, J.H.; Bakr, O.M.; Wu, T. Schottky junctions on perovskite single crystals: light-modulated dielectric constant and self-biased photodetection. *Adv. Electron. Mater.* **2016**, *4*, 8304-8312.
7. Tong, G.; Geng, X.; Yu, Y.; Yu, L.; Xu, J.; Jiang, Y.; Sheng, Y.; Shi, Y.; Chen, K. Rapid, stable and self-powered perovskite detectors via a fast chemical vapor deposition process. *RSC Adv.* **2017**, *7*, 18224-18230.
8. Tang, F.; Chen, Q.; Chen, L.; Ye, F.; Cai, J.; Chen, L. Mixture interlayer for high performance organic-inorganic perovskite photodetectors. *Appl. Phys. Lett.* **2016**, 109, 123301.
9. Sutherland, B.R.; Johnston, A.K.; Ip, A.H.; Xu, J.; Adinolfi, V.; Kanjanaboos, P.; Sargent, E. Sensitive, fast, and stable perovskite photodetectors exploiting interface engineering. *ACS Photonics* **2015**, *2*, 1117-1123.
10. Bao, C.; Zhu, W.; Yang, J.; Li, F.; Gu, S.; Wang, Y.; Yu, T.; Zhu, J.; Zhou, Y.; Zou, Z. Highly flexible self-powered organolead trihalide perovskite photodetectors with gold nanowire networks as transparent electrodes. *ACS Appl. Mater. Interfaces* **2016**, *8*, 23868-23875.
